# Re-thinking of the adhesion mechanism of Serine-aspartate repeat-containing protein D (SdrD)

**DOI:** 10.1063/4.0001142

**Published:** 2025-10-27

**Authors:** Younhchang Kim, Angela Tan, Priyanka Gade, Mike Enders, Xiaobing Zuo, Kemin Tan, Andrzej Joachimiak

**Affiliations:** 1 Argonne National Laboratory, Lemont, Illinois, USA; 2 University of Massachusetts Amherst, Amherst, Massachusetts, USA

## Abstract

Serine-aspartate repeat-containing protein D (SdrD) is a cell wall-anchored, calcium-binding protein of *Staphylococcus aureus*. It is a member of Sdr subfamily of the microbial surface components recognizing adhesive matrix molecule (MSCRAMM) family. SdrD plays a crucial role in bacterial adhesion and pathogenesis, contributing to a wide range of infectious diseases, including skin and soft tissue infections, in both healthcare facilities and community settings. Although several Sdr structures, including complexes with peptides, have been determined, the substrate and substrate binding mechanism of SdrD remain elusive.

Recently, we have determined a new crystal structure of SdrD and measured its solution small-angle X-ray scattering (SAXS). Structural analysis and comparison with existing Sdr structures as well as solution structural modelling have enhanced our understanding of this bacterial adhesin and its mechanism of molecular attachment to host cell.

I

